# Antimicrobial Features of Organic Functionalized Graphene-Oxide with Selected Amines

**DOI:** 10.3390/ma11091704

**Published:** 2018-09-13

**Authors:** Irina Zarafu, Ioana Turcu, Daniela C. Culiță, Simona Petrescu, Marcela Popa, Mariana C. Chifiriuc, Carmen Limban, Alexandra Telehoiu, Petre Ioniță

**Affiliations:** 1 Biochemistry and Catalysis, Department of Organic Chemistry, Faculty of Chemistry, University of Bucharest, 030018 Bucharest, Romania; zarafuirina@yahoo.fr (I.Z.); oana_turcu@yahoo.com (I.T.); 2 Institute of Physical Chemistry, 202 Spl. Independentei, 060021 Bucharest, Romania; danaculita@yahoo.co.uk (D.C.C.); simon_pet@yahoo.com (S.P.); 3 Faculty of Biology, University of Bucharest, 030018 Bucharest, Romania; bmarcelica@yahoo.com (M.P.); carmen_balotescu@yahoo.com (M.C.C.); 4 Research Institute of the University of Bucharest, ICUB, 050095 Bucharest, Romania; 5 Department of Pharmaceutical Chemistry, Carol Davila’ University of Medicine and Pharmacy, 020956 Bucharest, Romania; carmen_limban@yahoo.com (C.L.); alexandratelehoiu@yahoo.com (A.T.)

**Keywords:** graphene oxide, synthesis, functionalization, amines, antibacterial, anti-biofilm

## Abstract

(1) Background: Graphene oxide is a new carbon-based material that contains functional groups (carboxyl, hydroxyl, carbonyl, epoxy) and therefore can be easily functionalized with organic compounds of interest, yielding hybrid materials with important properties and applications. (2) Methods: Graphene oxide has been obtained by a modified Hummers method and activated by thionyl chloride in order to be covalently functionalized with amines. Thus obtained hybrid materials were characterized by infrared and Raman spectroscopy, elemental analysis and scanning electron microscopy and then tested for their antimicrobial and anti-biofilm activity. (3) Results: Eight amines of interest were used to functionalize grapheme oxide and the materials thus obtained were tested against Gram-negative (*Escherichia coli*, *Pseudomonas aeruginosa*) and Gram-positive (*Staphylococcus aureus*) bacterial strainsin plankonic and biofilm growth state. Both amines, as well as the functionalized materials, exhibited anti-microbial features. Three to five functionalized graphene oxide materials exhibited improved inhibitory activity against planktonic strains as compared with the respective amines. In exchange, the amines alone proved generally more efficient against biofilm-embedded cells. (4) Conclusions: Such hybrid materials may have a wide range of potential use in biomedical applications.

## 1. Introduction

Carbon-based materials are important pillar of development in human history, starting as an energy source and progressing on to high-tech devices. Graphite, an allotrope of carbon, consisting of a specific three-dimensional (3D) hexagonal arrangement of carbon atoms, can be converted into a 2D material by physical or chemical means. These 2D materials are currently known as graphene, which consist of a layer of carbon atoms arranged in a polycyclic aromatic structure. Graphene has unique physical, electrical, mechanical, and chemical properties, and constitutes a platform for advanced technologies [1,2,3].

Graphene oxide is a similar material that contains oxygen atoms as well in the form of organic functional groups (carboxyl, hydroxyl, carbonyl, epoxy). New organic compounds of interest can be covalently attached to these functional groups, yielding a hybrid or composite nanomaterial [4,5,6,7,8]. The functionalization of graphene oxide with biologically active molecules is a promising direction for the development of new materials with important applications, including medicinal ones.

Bacterial infections are one of the top public health problems worldwide, and the problem of antibiotic resistance exemplifies the issue in the post-antibiotic era, with severe medical consequences on mortality rates (especially in the infant population), failure of medical procedures (transplant, chemotherapy, implantology, etc.) and increasing costs [9]. In order to overcome antimicrobial resistance and the innovation gap in the development of novel classes of antibiotics, alternative strategies have been proposed, which include the use of different nanomaterials. Due to their large-spectrum biocidal activity, mediated by the multi-level interaction with different microbial structures (such as membrane, proteins and DNA), and low probability to resistance, graphene-based materials are promising candidates for the development of novel antibacterial agents and surfaces [10,11,12,13,14].

Graphenes have been used to disperse, stabilize, and deliver different nanomaterials or drugs, including antibiotics. In addition, due to their good biocompatibility, graphene-based materials have a wide panel of antimicrobial applications, such as antibacterial packaging, wound dressing, and disinfection [15].

The quest for new organic molecules with reliable biological activity remains a major topic in medicinal chemistry. Known molecules are also being tested for biological applications. Nitrogen containing compounds like amines and their derivatives (such as amides) are well-known bioactive compounds; for example, aminoacids, peptides, hormones or alkaloids are important [16,17], naturally occurring substances or medicines.

The attachment of bioactive compounds on materials can offer several advantages, such as higher stability of the active compound, good dispersion, protection against some environmental factors, and possibility of a controlled release, among others. Moreover, antimicrobial surface technologies are of a paramount importance in the development of new materials for implants or other medical uses [18].

**Figure 1:**
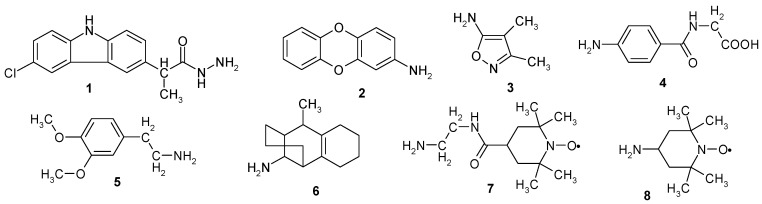
Chemical structures of the amines used in this study.

In this work, we first obtained graphene oxide, which was subsequently functionalized with several amine type organic compounds and the hybrid materials were tested as antimicrobial materials. The organic compounds used for functionalization are shown in Figure 1 (compounds **1**–**8**); all of them contain an amino group, necessary to form a covalent bond to the graphene oxide.

## 2. Materials and Methods

All chemicals and solvents were purchased from Sigma-Aldrich or Chimopar and used as received. Infrared (IR) spectra were recorded under normal conditions on a Jasco FTIR 4100 apparatus (KBr discs, Jasco Corporation, Tokyo, Japan). Elemental analysis was performed on a CHNPerkin Elmer 2400 apparatus (Perkin Elmer, San Jose, CA, USA). The morphology of the samples was investigated by Scanning Electron Microscopy (SEM) using a high-resolution microscope, FEI Quanta 3D FEG model (FEI, Brno, Czech Republic), operating in high vacuum mode with an accelerating voltage of 10 and 15 kV. Minimal sample preparation consisted of immobilizing the material on a double-sided carbon tape, without coating. Raman spectra were measured using a Horiba Jobin-Yvon LabRam spectrometer (Horiba SAS, Villeneuve d-Asq, France). Samples were excited with a 632.8 nm laser through an ×50LWD air objective of an Olympus microscope (Horiba SAS, Villeneuve d-Asq, France). Raman signal was energy-dispersed by a 600-line/mm groove density grating. Lorenzian fitting of the background corrected spectra was carried out by means of a PeakFit 4.12 software.

### 2.1. Graphene Oxide Synthesis and Functionalization

The synthesis of graphene oxide was carried out following the improved method described by Tour [19], with slight modifications as follows: 1 g of graphite and 6 g of potassium permanganate was carefully mixed with a solution formed from 135 mL concentrated sulfuric acid and 15 mL concentrated phosphoric acid. The mixture was stirred for about 10 h at a temperature of about 50 °C (achieved by external heating) and then poured on about 200 g of ice; after the ice melted, distilled water was added to a final volume of 1 L, and hydrogen peroxide (30%) was added until the violet solution turned yellow (about 10 mL). The mixture was left to settle overnight and the next day the supernatant was decanted. Diluted hydrochloric acid (1 M) was added (500 mL) to the slurry and the mixture stirred for 15 min and then left to settle again for a few hours. The procedure was repeated three times and then the hydrochloric acid replaced by 200 mL methanol; this step was also repeated thrice. Finally, the solid was separated by centrifugation and left in open air to dry, followed by advanced drying at 60 °C under vacuum for 1 h. The solid was later ground using a pestle.

Functionalization of graphene oxide was achieved as previously described [6]. In short, 0.2 g of graphene oxide were suspended in 10 mL dry dichloroethane, to which 1 mL thionyl chloride and 3 drops of DMF (dimethylformamide) were added. The mixture was refluxed for 2 h, the solid decanted, washed with DCM (dichloromethane) and then re-suspended in 10 mL DCM to which each amine was added (200 mg), followed by 1 mL of triethylamine. The mixture was left overnight and the next day the solid was separated, washed with DCM and methanol, and then dried.

### 2.2. In Vitro Antimicrobial Activity Assay

The antibacterial activity of the synthesized materials—abbreviated **GO1IZ**–**GO8IZ**—of the respective amines and of the unfunctionalized GO was assessed on Gram-negative (*Escherichia coli* ATCC 25922 *Pseudomonas aeruginosa* ATCC 25923) and Gram-positive (*Staphylococcus aureus* ATCC 25923) bacterial strains, using tetracycline (TCY) as a standard antibiotic. The microbial suspensions of 1.5 × 10^8^ CFU mL^−1^ (0.5 McFarland density) were obtained from fresh culture obtained in triptic soy agar incubated at 37 °C for 24 h.

Quantitative analysis of the antibacterial activity was performed by broth microdilution method in 96 multi-well plates and allowed to establish minimum inhibitory concentration (MIC) values for the obtained compounds. Each well containing binary dilutions of compound solutions, starting from 5 to 0.008 mg/mL, and 100 μL volume of broth, was seeded with 20 μL microbial inoculum, reaching a final density of 10^5^ CFU mL^−1^. Thereafter, the plates were incubated for 24 h at 37 °C, and the MIC values were considered as the lowest concentration of the tested samples, inhibiting visible growth of the bacterial culture [20].

Following the MIC assay, anti-biofilm activity was assessed by the crystal violet microtiter assay. The following steps were taken: The content of the plates was removed after reading of the MICs, the plates were washed three times by phosphate buffered saline, the biofilms adhered to the plastic walls were fixed with cold methanol, the fixed biofilms were stained by crystal violet solution for 15 min and finally the colored biofilms were resuspended in a 33% acetic acid solution. The density of the microbial biofilm harvested from the plastic wells was measured by reading the optical density at 490 nm for the coloured suspensions. The minimal biofilm eradication concentration (MBEC) value corresponded to the concentration found in the well in which the absorbance values were inferior to those of the positive control [21]. All assessment were performed in triplicates and the data were analyzed with the GraphPad Prism version 5.00 for Windows, La Jolla California USA, www.graphpad.com.

## 3. Results and Discussion

### 3.1. Synthesis

Graphite is the standard material to obtain graphene oxide. The classical procedure involves the employment of a strong oxidation mixture (i.e., potassium permanganate in concentrated sulfuric acid) that initiates the formation of oxygen-rich functional groups, like carboxylic groups, etc. [22]. During the oxidation procedure, as well as due to the thermal processes that accompany this and the work-up procedure, exfoliation occurs, usually yielding the so-called few-layer graphene oxide (preparation of single-layer graphene oxide requires extensive sonication).

**Figure 2:**
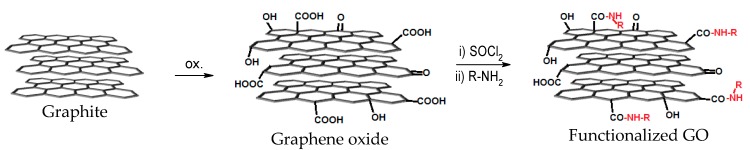
Synthesis and functionalization of graphene oxide with amines.

To covalently link our compounds of interest to the graphene oxide, we have chosen the path of amide group formation (between the carboxylic groups from graphene oxide and the amino group from the organic compounds). Thus, the carboxylic groups are activated by thionyl chloride (yielding the corresponding acid chloride) and these easily react with amines (that act as a nucleophile) [6]. In this way, the organic compounds are covalently attached to the graphene oxide via an amide link (Figure 2).

The amines used have some important properties, i.e., compounds **1**–**3** (Figure 1) are hetero-cycles [23] known for their biological activity (compound **1** is a non-steroidal anti-inflammatory drug known under the name of Caprofen). Compounds **7** and **8** are stable free radicals that have important redox properties [6]. Free radicals are often encountered in degenerative diseases [24,25].

### 3.2. Structural Analysis

The final materials obtained by covalent coupling of the organic compounds **1**–**8** to the graphene oxide were noted from **GO1IZ** to **GO8IZ** and were characterized by elemental analysis, infrared (IR) and Raman spectroscopy and scanning electron microscopy (SEM).

**Figure 3:**
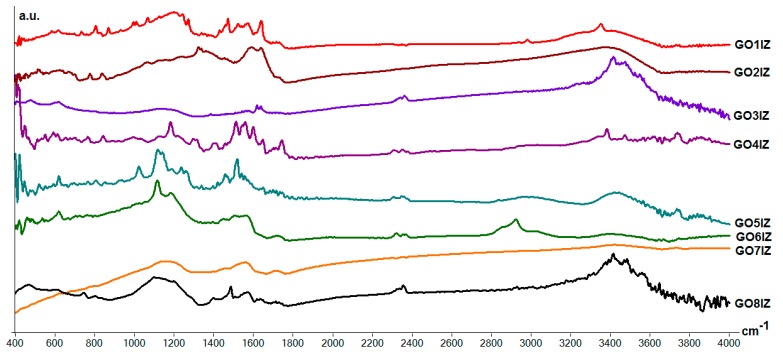
IR spectra of the materials **GO1IZ**–**GO8IZ.**

IR spectra of the hybrid **GO1IZ**–**GO8IZ** materials are showed in Figure 3. As a general feature, it was noticed that all the spectra show several intense bands between 1500 and 1700 cm^−1^; these are mainly attributed to carbonyl groups that are present as amide groups. Elemental analysis for all samples showed a nitrogen content of about 2.3–4.1%, which is proof of the presence of the organic component (amide) in the graphene oxide (**GO1IZ** 4.06%, **GO2IZ** 2.37%, **GO3IZ** 3.33%, **GO4IZ** 2.77%, **GO5IZ** 2.43%, **GO6IZ** 3.12%, **GO7IZ** 2.87%, **GO8IZ** 3.56% nitrogen content). In addition, in the same region of 1500–1700 cm^−1^ some bands should be attributed to a number of free carboxyl end groups, as well as from aromatic C=C bonds. Other important bands are noticed around 1100–1200 cm^−1^ and 3400 cm^−1^, and these are attributed to C–O and –OH vibrations, respectively.

**Figure 4:**
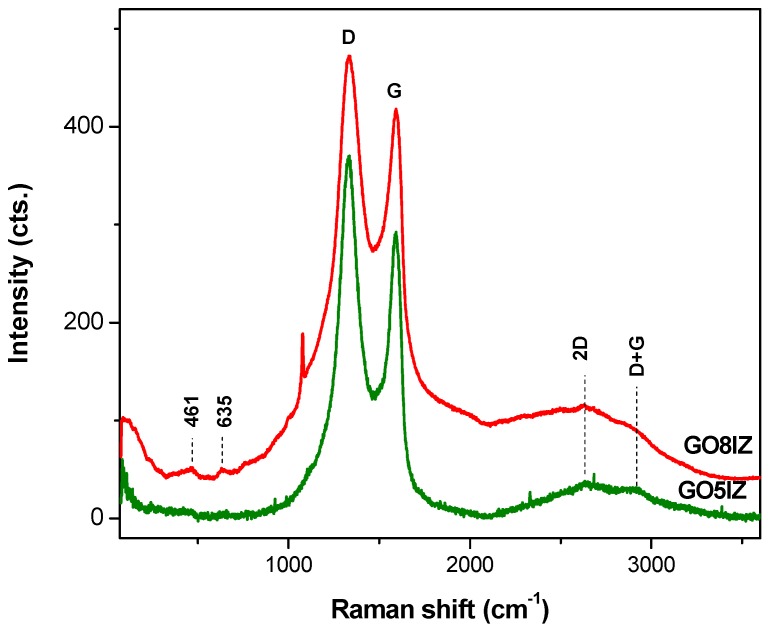
Raman spectra of the functionalized graphene oxides (**GO5IZ** and **GO8IZ** samples).

Raman spectra showed the well-known *D* and *G* peaks of the sp^2^-hybridized carbon atoms in graphene oxide (Figure 4), at values of 1330 cm^−1^ and 1590 cm^−1^, respectively. The *D* band (or the disorder band) corresponds to the disruption of the sp^2^-bonded lattice of graphite by the formation of carbon-oxygen bonds in the graphene oxide samples, leading to the distortion and opening of the aromatic rings. The *G* band corresponds to the vibrations of the sp^2^ carbon in the graphite lattice [26]. The intensity ratios between these Raman bands give values higher than 1, which means that the crystallite domains are reduced. Although widely used for characterization of the graphene oxide materials, this ratio is not entirely reliable [27,28]. Instead, modifications of the *D*, *G* and *2D* bands give evidence of graphene oxide functionalization [29]. Additional bands are present in functionalized graphene oxides; moreover, the high wavenumbers (containing *2D*, *D+G* bands) might obscure hydrogen bonding in the amides, formed by functionalization of graphene oxides [30].

**Figure 5:**
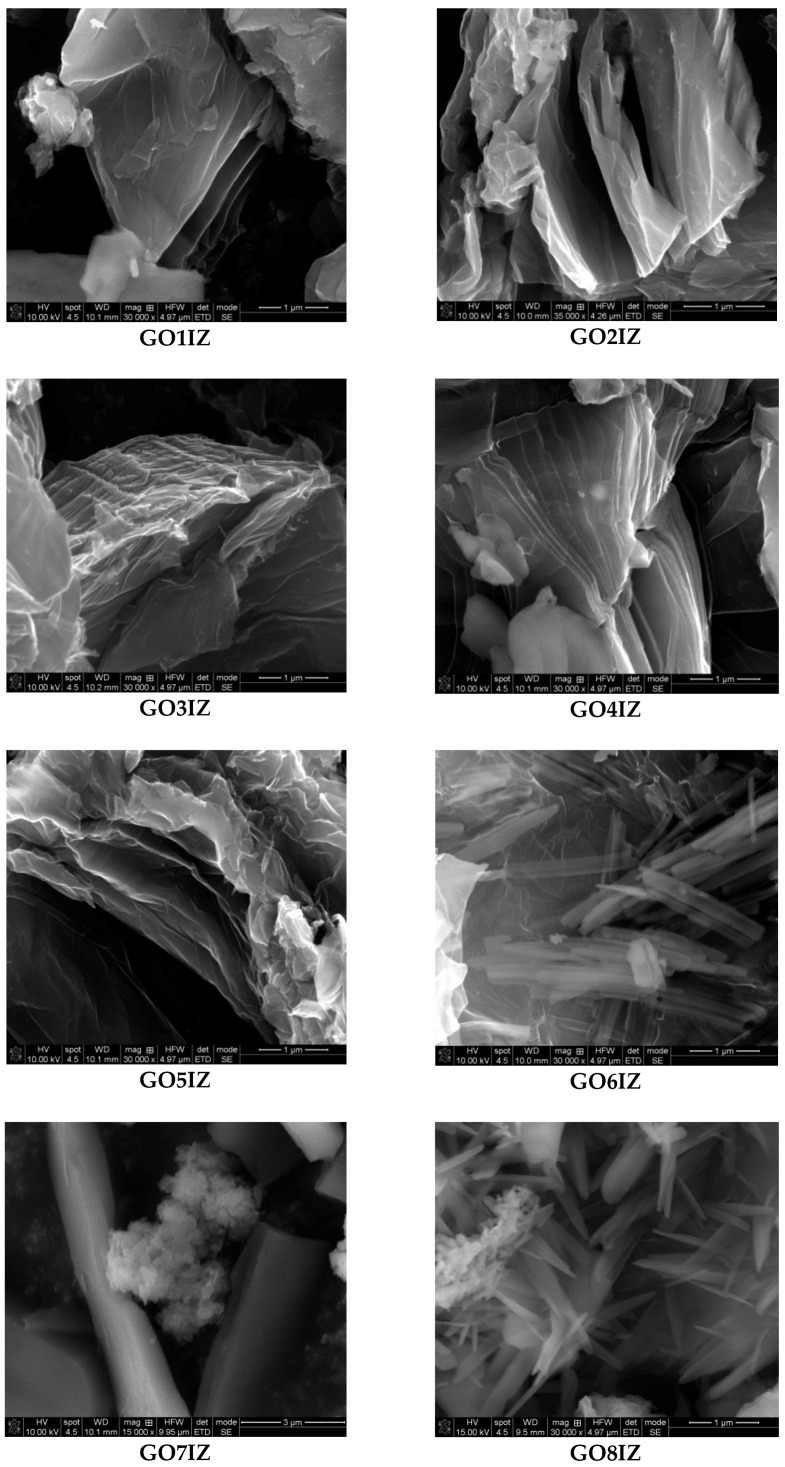
SEM pictures of samples **GO1IZ**–**GO8IZ**.

SEM was performed to confirm the morphology of the samples; the images are shown in Figure 5 and Figure S1. The nanostructure of the functionalized graphene oxide is presented as stacked flakes. The arrangement of the graphene oxide layers is visible in all the samples. The materials are composed of flat flakes with fairly straight edges that can easily delaminate. However, samples **GO6IZ** and **GO8IZ** present some differences—they look more like nanotubes, probably obtained from graphene oxide delaminated sheets by a rolling-up process (induced by some possible interactions not further investigated).

### 3.3. Biological Assessment

**Figure 6:**
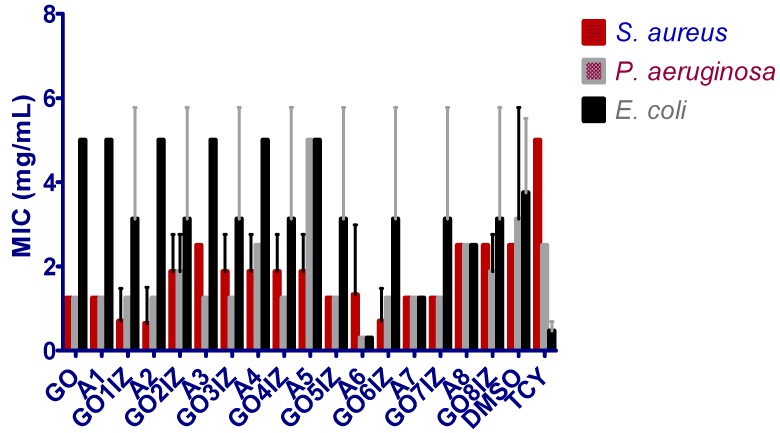
MIC values obtained for the synthesized compounds against the tested strains.

Due to the increasing problem of bacterial resistance, finding new antibacterial agents or combinations is a top research priority. The antimicrobial features of graphene oxide extend towards multi-resistant strains, pathogenic not only in humans [31,32], but also in bacterial and fungal phytopathogens [33], demonstrating the high potential of GO for antimicrobial applications, not only in the biomedical field, but also in agriculture. It has been shown that GO could also be used as a carrier for antibiotics [13,34]. It is generally accepted that the local tissue changes induced by the occurrence of inflammation could limit the in vivo efficiency of different antibiotics. Therefore, in this paper, we have exploited the concept of multicomponent therapy, by coupling an anti-inflammatory amine with GO, in order to improve the biocompatibility of GO as well as obtain a multi-target antibacterial agent, efficient against planktonic and biofilm—embedded bacterial cells that are less prone to be inactivated by or to select antibiotic drug resistance mechanisms. With a few exceptions, the tested compounds proved to be the most efficient against *P. aeruginosa* strain(Figure 6). Only three of the tested compounds—**A2**, **GO1IZ** and **GO6IZ**—proved to be more efficient against the *S. aureus* strain than the other two strains. Excepting **A2** and **TCY**, the tested compounds proved to be less efficient against the *E. coli* strain in planktonic growth, as compared to the other two strains (Figure 6). The comparative evaluation of the functionalized graphenes’ antimicrobial activity, in comparison to that of the corresponding amines, revealed a superior inhibitory activity of the planktonic growth of *S. aureus* strain for **GO1IZ**, **GO3IZ**, **GO5IZ**, **GO6IZ**, of the *P. aeruginosa* strain for **GO4IZ**, **GO5IZ** and **GO8IZ**, and of the *E. coli* strain for **GO1IZ**, **GO2IZ**, **GO3IZ**, **GO4IZ** and **GO5IZ**.

**Figure 7:**
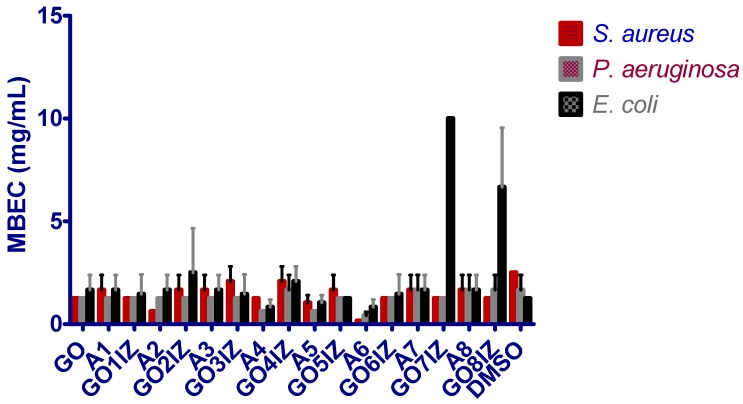
MBEC values obtained for the synthesized compounds against the tested strains.

The anti-biofilm behavior of the obtained substances was much more nuanced, depending on the tested strain (Figure 7). As expected, taking into account the high phenotypic resistance of microbial biofilms to different limitative conditions, including the effect of antimicrobial substances, the MBEC values were in some cases higher than the MIC ones. Concerning the efficiency of the tested compound against the three strains, very high anti-staphylococcal biofilm efficiency of **A2**, **A6**, **A8**, efficiency of **A4**, **A5** and **A6** against *Ps. aeruginosa*, and of **A4** and **A6** against *E. coli* biofilm was noticed.

Out of the three tested strains, the biofilm formed by *Ps. aeruginosa* was the most susceptible. The majority of the tested compounds, excepting **A2**, **A6** and **GO8IZ**, exhibited lower MBEC values, as compared to those obtained for the *S. aureus* strain. The comparative evaluation of the functionalized graphenes’ antibiofilm activity in comparison to that of the corresponding amines revealed a superior inhibitory activity of **GO1Z** and of **GO8IZ** against *S. aureus* biofilm development, and of **GO7IZ** against *S. aureus* and *P. aeruginosa* biofilms.

Taken together, the results of the quantitative assays of the antimicrobial activity of the tested compounds demonstrated that some of the functionalized graphenes exhibited improved antimicrobial properties as compared to GO and the corresponding amines being active against planktonic and biofilm-embedded microbial cells.

## 4. Conclusions

In this study, we combined the advantages of graphene oxide, already known for its large antimicrobial spectrum, with those of amides with anti-inflammatory activity in order to obtain improved antimicrobial systems efficient against planktonic bacteria and biofilms.

Functionalization with organic compounds of nanostructured graphene oxide has been proven and the biological assessment of their antimicrobial properties has demonstrated that at least three hybrid materials showed better antimicrobial activity as compared to the corresponding amine against each tested bacterial strain in the planktonic growth state. However, in the case of bacterial biofilms, amines have shown better inhibitory activity, as compared to the hybrid systems, excepting **GO1Z** and of **GO8IZ** for *S. aureus* and **GO7IZ** for both *S. aureus* and *P. aeruginosa* biofilms. The obtained results are encouraging and show the potential of the obtained hybrid materials to be used in a wide range of antimicrobial applications, both in the biomedical and agricultural fields.

## Supplementary Materials

The following are available online at http://www.mdpi.com/1996-1944/11/9/1704/s1. Figure S1: Additional SEM picture of the samples **GO1IZ**–**GO8IZ**.
