# Supplementary material for: Antimicrobial Features of Organic Functionalized Graphene-Oxide with Selected Amines
Source: Materials (Basel). 2018 Sep 13;11(9):1704. doi: 10.3390/ma11091704 (PMC6164380; doi:10.3390/ma11091704)
Supplement: Supplementary file 1 [file materials-11-01704-s001.zip › materials-352701-SI.pdf]

Supplementary Materials: Antimicrobial Features of Organic Functionalized Graphene-Oxide with Selected Amines

Irina Zarafu [1], Ioana Turcu [1], Daniela C. Culiță [2], Simona Petrescu [2], Marcela Popa [3,4], Mariana C. Chifiriuc [3,4], Carmen Limban [5], Alexandra Telehoiu [5], Petre Ioniță [1,2,*]

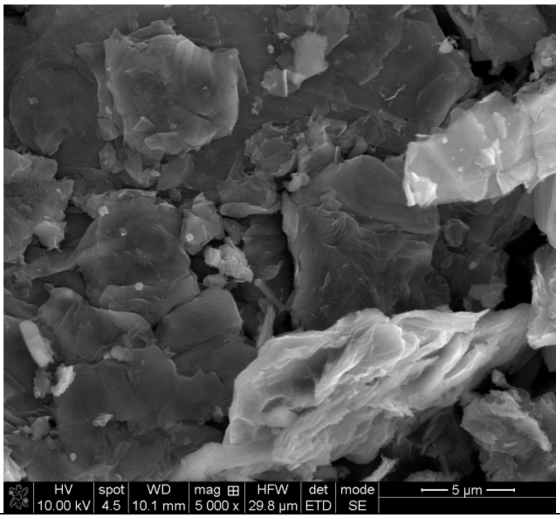

GO1IZ

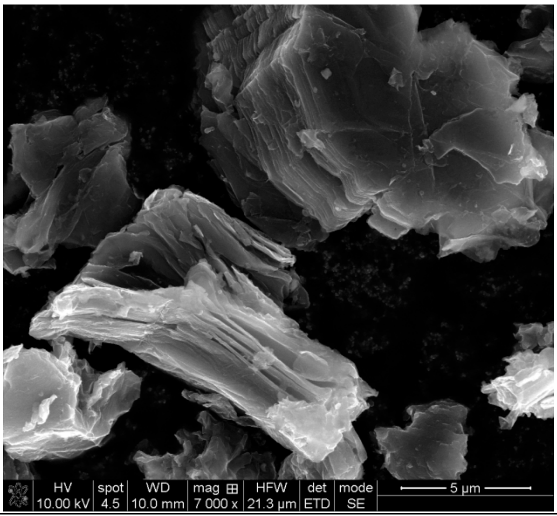

GO2IZ

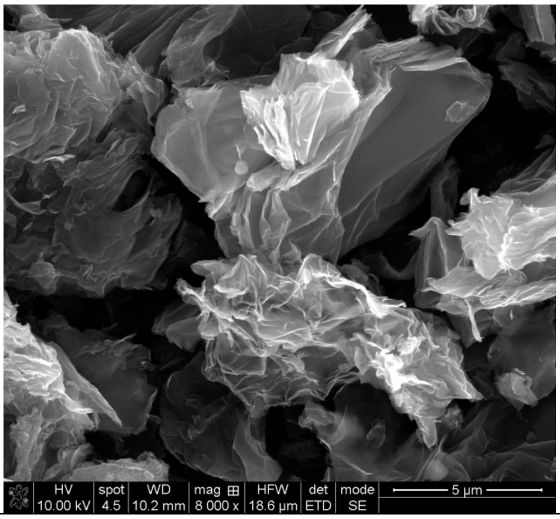

GO3IZ

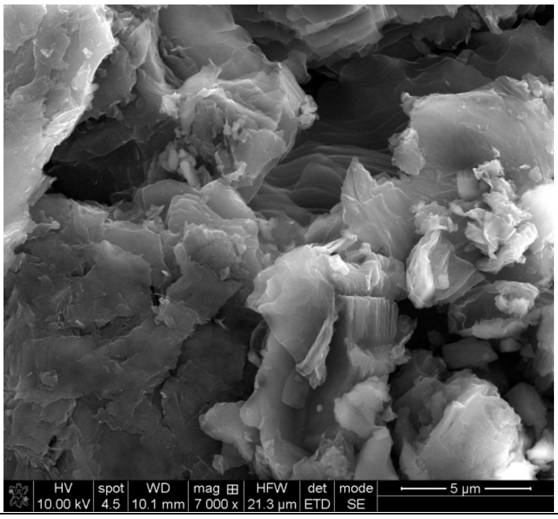

GO4IZ

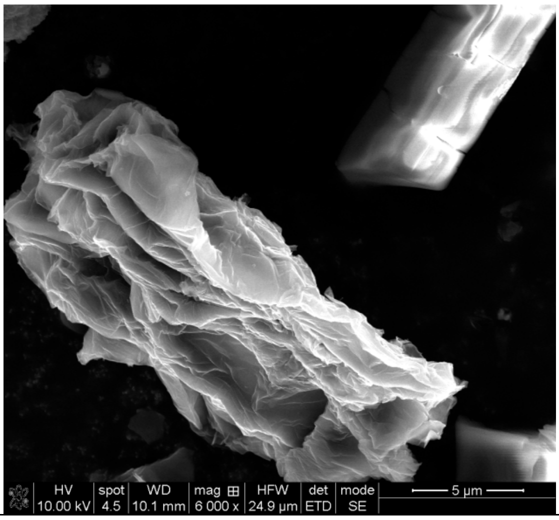

GO5IZ

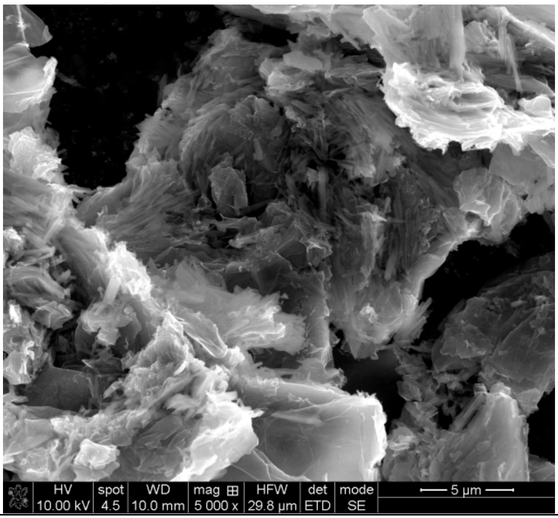

GO6IZ

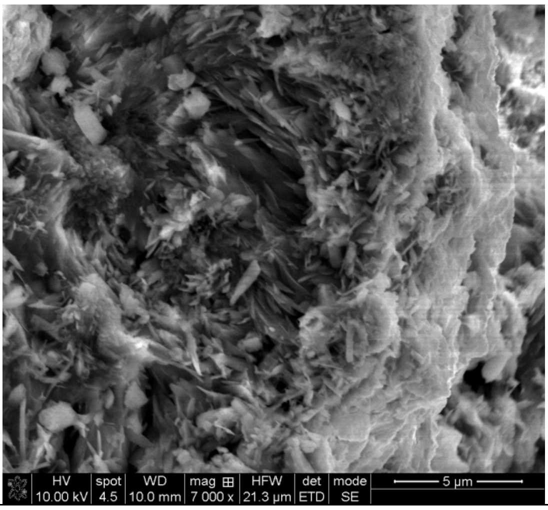

GO7IZ

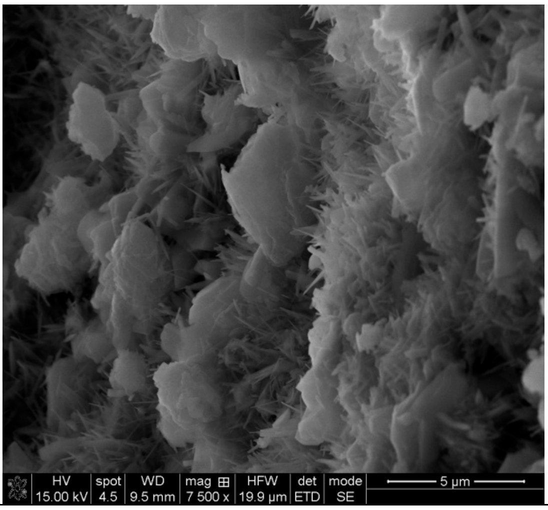

GO8IZ

Figure S1. Additional SEM pictures of samples **GO1IZ–GO8IZ.**
